# Role of Imaging in the Evaluation of Minimal Residual Disease in Multiple Myeloma Patients

**DOI:** 10.3390/jcm9113519

**Published:** 2020-10-31

**Authors:** Elena Zamagni, Paola Tacchetti, Simona Barbato, Michele Cavo

**Affiliations:** Dipartimento di Medicina Specialistica, Diagnostica e Sperimentale, Istituto di Ematologia “Seràgnoli”, Università degli Studi, Azienda Ospedaliero-Universitaria di Bologna, 40138 Bologna, Italy; paola.tacchetti2@unibo.it (P.T.); simona.barbato3@unibo.it (S.B.); michele.cavo@unibo.it (M.C.)

**Keywords:** PET/CT, MRI, myeloma, prognosis, minimal residual disease

## Abstract

The International Myeloma Working Group (IMWG) recently introduced the evaluation of minimal residual disease (MRD) within the multiple myeloma (MM) response criteria, and MRD negativity assessed inside and outside the bone marrow is currently considered the most powerful predictor of favorable long-term outcomes. However, MRD evaluation has thus far relied on flow-cytometry or molecular-based methods, despite the limitations associated with the patchy infiltration of bone marrow (BM) plasma cells and the presence of extra-medullary (EMD). On the contrary, imaging-based sensitive response assessment through the use of functional rather than morphological whole-body (WB) imaging techniques, such as positron emission tomography with computed tomography (PET/CT) and magnetic resonance imaging (MRI), likely is a promising strategy to overcome these limitations in evaluating response to therapy and in the assessment of the MRD status in MM patients. However, despite the significant advances in the development and availability of novel functional imaging techniques for MRD evaluation, a worldwide standardization of imaging criteria for acquisition, interpretation, and reporting is yet to be determined and will be object of future investigations.

## 1. Imaging for Minimal Residual Disease (MRD) Evaluation

In 2016, the International Myeloma Working Group (IMWG) formally introduced the evaluation of minimal residual disease (MRD) within the multiple myeloma (MM) response criteria [1]. MRD is usually assessed in the bone marrow (BM) by means of cellular-based (flow-cytometry) or molecular-based (next-generation sequencing) methods. Since plenty of data correlate the depth of response with long-term outcomes, information on MRD may represent a valid and early biomarker for the efficacy of treatment [2]. However, BM plasma cell (BMPCs) infiltration is often irregular, likely increasing the probability of a false-negative evaluation by using techniques relying on BM specimens—by nature limited to a very small area of the body—with no identification of extra-medullary (EMD) escape in case of metastasis [3]. This phenomenon is currently quite frequent, likely depending on extended overall survival (OS) and the increasingly massive use of functional imaging techniques, and usually culminates in a fatal clinical outcome, despite the progress made with novel therapeutic approaches [3,4,5,6].

Besides the patchy infiltration of BMPCs and the presence of EMD, recent prospective studies monitoring patients with serial functional imaging and focal lesion (FL) biopsies have demonstrated that MM implies a great spatial heterogeneity which is proportional to the size of a FL, and different disease clones with different genomic profiles may coexist in BM and FLs [7,8].

For the longest time, imaging in myeloma was based on skeletal survey and limited at staging or re-staging to determine myeloma bone disease. However, this technique is not very sensitive, partly because the healing of bone is limited [9], but also because it is ineffective on soft tissues/masses, being therefore unusable in assessing response to therapy. In such cases, morphological whole-body (WB) imaging techniques should be replaced by functional evaluations, such as positron emission tomography with computed tomography (PET/CT) and magnetic resonance imaging (MRI), both providing a global representation of the tumor burden beyond osteolytic lesions and showing further prognostic markers such as EMD [10]. Patients carrying soft plasmacytomas, either extra-medullary (5–10% at diagnosis, higher percentage in the later phases of the disease) or paramedullary (20–30%), more often display a discrepancy between the presence of EM sites of clonal proliferating PCs upon BM MRD negativity [11].

## 2. 18F-FDG-PET/CT

One of the best imaging techniques by which to assess tumor metabolic activity and determine treatment efficacy is ^18^Fluorine-fluoro-deoxyglucose (18F-FDG) PET/CT [12,13]. The wide use of this tool mostly relies on its capability of distinguishing between active and inactive (e.g., fibrotic) disease and by the fact that low-dose CT, typically associated with FDG-PET for localization, can precisely map the sites of bone and extra-medullary disease [12].

### 2.1. Methods and Standardization

Largely used worldwide to evaluate and monitor metabolic response to therapy, the 18-FDG PET/CT technique is quite standard. With 18F-FDG as the most used tracer, whose dose administration slightly varies based on the system and on the patient’s weight, image acquisition and reconstruction follow standard procedures, as well as patient preparation [12], with a total procedure time of approximately 80–90 min. At least the skull, upper limbs, and femurs should be included in the field of view (FOV) of 18F-FDG PET/CT, extended to the lower limbs under certain circumstances. The attenuation, correction, and interpretation of the image are achieved by CT at low doses (120 kV, 80 mA). Hypermetabolic bone lesions, irrespective of underlying lytic lesions at CT, are identified with a PET standard spatial resolution limit of approximately 5 mm, even in patients with kidney disease and/or metallic bone implants. Multiplanar cuts are obtained through an ad hoc workstation upon image reconstruction.

Image interpretation typically relies on the standardized uptake value (SUV) maximum (max), especially to evaluate the efficacy of therapy after treatment. This is a semi-quantitative index, traditionally indexed to the background on L1/L2, if healthy; the liver; the spleen; or the mediastinal blood pool (MBP). However, there is as yet no standardization for scan evaluation in MM. Besides this, some other parameters may be considered in MM, such as the Total Metabolic Tumor Volume, with this parameter being an index of the total volume of active disease in the PET FOV, and the total lesion glycolysis, reflecting the glycolytic phenotype of FLs.

To gain a PET negativity, according to the IMWG definition, each increase in tracer uptake at baseline or at previous PETs/CTs should disappear, or become inferior to the SUV of the MBP or of the surrounding normal tissue [1]. However, since many factors may influence the SUV_max_ (first of all the uptake time), an attempt to standardize the cut-offs for positivity/negativity and achieve criteria for metabolic response has been made [14]. In this sense, previous findings revealed that the value of Deauville scores (DS), tested for the first time in MM [15], can be used for this purpose. Indeed, with this value being representative of the outcomes of the various patients, it has been proposed to define the PET complete metabolic response (CMR) after therapy, using the liver as a background (DS 4). The proposed PET metabolic response criteria are listed in Table 1.

When evaluating the 18F-FDG PET/CT imaging scans, false-positive/negative results may arise and should be excluded from the analysis. In particular, patients lacking the hexokinase enzyme (10–15%) may have no 18F-FDG-avid PCs, [16], since this enzyme mediates FDG trapping in the cells; in such patients, FDG PET after treatment is not recommended [10,13], and an alternative tracer and/or tool should be considered. Moreover, false-negative results can be obtained soon after the use of high-dose steroids, due to the transient profound suppression of tumor metabolism and the competitive inhibition of FDG uptake from plasma cells related to increased glucose levels. On the contrary, the recent use of chemotherapy and/or growth factors, inducing bone marrow reconstitution, may result in false-positive findings. For these reasons, when possible, PET/CT should be performed at least one month after the use of all these agents.

Beyond 18F-FDG, preliminary investigations [17] have been carried out to test the sensitivity and specificity of new PET/CT tracers, targeting alternative metabolic pathways or different PC receptors that might prove to be valid molecular imaging biomarkers. PET imaging targeted to CXCR4 [18,19] and CD38 [20,21] has advanced into translational clinical trials, bringing us closer to powerful imaging options for myeloma. Antigenic-expressing tumor cells may be visualized through the use of radiolabelled antibodies, independently of metabolic processes, to obtain early and more accurate response assessments. For example, all MM cells express CD38, making them an excellent focus for targeted imaging and therapy. Daratumumab is an FDA-approved monoclonal antibody therapy for multiple myeloma that targets CD38. Conjugating daratumumab with the positron emitting radio-isotopes Copper-64 (64Cu) and Zirconium-89 (89Zr) has allowed the creation of immunoPET tracers for myeloma imaging [22,23]. 89Zr-daratumumab has demonstrated the ability to detect multiple myeloma in early clinical trials [22] which was overlooked by FDG PET/CT and other clinically standard imaging methods. Copper-64-daratumumab has been demonstrated as well to provide a safe whole-body imaging of MM [23], and the comparison with FDG is currently on-going. More advanced clinical trials for these immunoPET agents are planned. At the moment, however, it is not possible to gain any definitive conclusion, since the availability of these new tracers is still limited and no prognostic data and/or standard reporting have been gathered, not to mention the high heterogeneity of tumors among patients in specific targets.

### 2.2. Clinical Studies

PET-positive lesions after therapy have been largely linked to unfavorable prognosis. Indeed, a number of studies reported that, in the case of complete remission (CR), patients with FDG-PET/CT negativity after ASCT had a lower risk of progression or death with respect to those with metabolically active sites of the disease [23,24,25,26,27,28,29]. Moreover, in patients achieving MRD negativity by flow cytometry (10^−5^ sensitivity), imaging—either by PET/CT or whole body-diffusion weighted imaging-magnetic resonance (WB-DWI-MRI)—was positive in 12% of the cases, attributing to these patients a worse prognosis in terms of progression-free survival (PFS) [30]. On the other hand, patients reaching CR with MRD negativity during salvage therapy often displayed FLs (50%). Additionally, it has recently been shown that patients obtaining PET FL normalization upon therapy have a comparable prognosis to those with no boosted metabolism at baseline, stressing the importance of prolonging the treatment until glucose metabolism is suppressed [28].

The complementarity between imaging (either FDG-PET/CT or WB-DWI-MRI) and BM techniques in defining the prognosis of patients was demonstrated by two prospective trials, applying flow cytometry as a BM technique with a sensitivity threshold of 10^−4^ [25] and 10^−5^ [30]. Moreover, very recently, the DS criteria to define PET/CT complete metabolic response have proven to be valid and applicable in newly diagnosed transplant-eligible MM (NDTEMM) patients, also confirming the complementarity with BM techniques [31].

On such a premise, 18F-FDG PET/CT currently is listed as the best imaging technique to evaluate and monitor metabolic response to therapy [12,13].

## 3. Magnetic Resonance Imaging (MRI)

The best tool to define the degree of BMPC infiltration, even prior to bone destruction, currently is the MRI, since this technique can visualize large volumes of BM and has a high sensitivity. As a result, this imaging technique is very useful in case of low tumor burden (e.g., in early stages or upon systemic therapy) [10,13,32]. Variations in MRI patterns may be attributable to the effects of therapy and may therefore be used to determine the efficacy of anti-myeloma treatment. In this sense, particular attention is given to MRI functional approaches, considered as the best option to evaluate disease after therapy, such as dynamic contrast-enhanced (DCE) and diffusion-weighted imaging (DWI) [33].

### 3.1. Methods and Standardization

MRI examines the water and fat content within tissue and is the most sensitive tool to detect the infiltration of BM in MM, with no radiation exposure [32,33]. An MM typical MRI pattern is the following: hypo-intensity in T1-weighted, hyper-intensity in T2-weighted with fat suppression in opposed phase imaging, and increased contrast-enhancement in T1-weighted sequences. Signal intensity is usually compared to the intervertebral disk as a reference. At least a 5 mm diameter is required to define a FL, also depending on the thickness of the MRI slice. The marrow involvement in MM may be classified into five different patterns: normal, focal, diffuse, combined focal, and diffuse and variegated or “salt and pepper” or variegate. The minimal FOV in MM is the axial skeleton (spine and pelvis), but more and more space is nowadays given to the whole-body protocol (WB-MRI). This technique is based on T1, T1 non-fat-saturated, and STIR sequences, usually not requiring contrast infusion; the total scan time for a whole-body image is generally less than 20 min. Although contrast agents are not always necessary, thanks to the high resolution of non-enhanced MRI for the BM, they are usually based on gadolinium, which is relatively inert, except in case of renal failure, where it can cause a nephrogenic systemic fibrosis. As a further extension of MRI, DWI measures the movement of water molecules in the tissue, without the need for a contrast medium. Limited movements indicate high cellularity; on the contrary, an increase in movement reflects low cellularity and/or increased microcirculation. Semi-quantitative parameters, such as the apparent diffusion coefficient (ADC), correlate with a higher or lower cellularity [34], with the correlation between ADC and histological infiltration by BMPCs being clearly demonstrated.

The process of acquisition, interpretation, and reporting of WB-MRI is very important, thus allowing the use of this technique to assess response to therapy; an attempt in this regard has very recently been proposed [35].

### 3.2. Clinical Studies

Conventional MRI without contrast agents has been used to assess response after therapy in several clinical trials as addition to serological and BM-derived parameters. Two studies using MRI of the spine and pelvis and whole-body MRI in a total of 711 patients treated with high-dose chemotherapy protocols showed that residual lesions after completion of the most aggressive part of treatment had a significant adverse prognostic significance [36,37]. A later study comparing again axial MRI (including spine and pelvis) in 134 patients with multiple myeloma with PET/CT in the same patients treated in a multi-center trial showed that PET/CT was superior to MRI with regard to prognostic significance after therapy. The most likely explanation for this has been already examined in the first study of this kind, showing that the treatment response in MRI appears delayed, with focal lesions of myeloma disappearing slower because MRI is not able to differentiate between vital and necrotic tissue within preexisting osteolytic lesions [36]. Interestingly, a change in lesions into a more liquid or cystic appearance was associated with a higher rate of complete remissions but also with a higher proliferation index in gene expression profiling [38]. A further development of MRI is the DWI approach [34] that allows us to assess cellularity and microcirculation in the BM [39]. Recent studies suggest that whole-body DWI might be equivalent or even slightly superior to PET/CT in the assessment of the residual disease of multiple myeloma [40,41,42,43,44,45,46]. However, thus far, the assessment of metabolic response by means of DWIMRI is yet limited by the absence of a uniform interpretation of the results because of the differences in microenvironment and cellularity, which are associated both with age and necrosis, the latter being caused by ongoing treatments. Additionally, the best timing to assess response is still a matter of debate. An interdisciplinary group of clinicians and radiologists is currently working on this aspect to overcome this limitation [35].

### 3.3. Choice of Imaging Technique for MRD Evaluation

Thus far, the evaluation and monitoring of response to therapy in MM is entrusted to FDG-PET/CT, which is considered the best imaging technique for correlating post-treatment findings with outcomes [1,10,12,13]. PET/CT is highly recommended in all patients that need to be tested for MRD after therapy. Indeed, this technique is capable of distinguishing cellular tissue from necrosis, which is crucial in these cases.

However, the FDG-based technique has some limitations, as discussed, and no homogeneous and prospective data are available on a comparison between FDG PRT/CT and WB-DWMRI in evaluating response to treatments. In case of negative imaging evaluation after therapy, serial evaluation until relapse can be recommended in patients with extra or para-medullary disease at diagnosis or in more advanced phases of the disease, when the risk of exclusive imaging-progression is higher. On the contrary, in patients with residual lesions, an annual follow-up can be recommended because of the high risk of early progression [29]; in such cases, functional imaging may be undoubtedly advantageous (Table 2).

## 4. Open Issues and Future Steps

Significant advances in MRD evaluation are based on the availability of novel functional imaging techniques, although many issues still need to be further investigated [47] (Table 3). Among these, it is an absolutely priority to achieve a complete standardization of guidelines for the acquisition, interpretation, and reporting of these imaging techniques, an attempt which is currently ongoing for both FDG PET/CT and WBMRI. Moreover, further investigation should be dedicated to the newer PET tracers and to DWIMRI, both needing to be correlated with clinical outcomes. DWIMRI images and PET/CT scans should also be prospectively compared, before and after therapy, to determine which technique is optimal for different patients and subgroups, and for different stages of disease both during and after treatment. Additionally and noteworthily, the concordance between complete metabolic response and flow or sequencing MRD negativity at the BM level need to be further explored, with different sensitivity thresholds (10^−5^–10^−6^), as well as the complementary role of imaging techniques in detecting MRD, within or outside the BM, with either cellular or molecular-based tools. Similarly, the role of imaging techniques in determining a sustained MRD, or its loss, remains to be clearly defined, along with the identification of the optimal time points at which to achieve such an assessment. Finally, the impact of MRD-driven treatment strategies has not been defined yet. Upcoming prospective trials that apply these new techniques extensively and evaluate MRD both inside and outside the BM will finally address these open issues and optimize the use of imaging in daily clinical practice.

## Figures and Tables

**Table 1:** Proposed refinement of the PET response criteria after therapy.

Pet Response after Therapy	Response Criteria
CMR (complete metabolic response)	Uptake ≤liver activity in BM sites and FLs previously involved (including extra-medullary and para-medullary disease) (DS1–3).
PMR (partial metabolic response)	Decrease in the number and/or activity of BM/FLs present at baseline, but persistence of lesion(s) with uptake >liver activity (DS 4 or 5).
SMD (stable metabolic disease)	No significant change in BM/FLs compared to baseline.
PMD (progressive metabolic disease)	New FLs compared to baseline consistent with myeloma.

Abbreviations: BM, bone marrow; DS, Deauville scale; FL(s), focal lesion(s).

**Table 2:** PET/CT and MRI in the evaluation of response to therapy.

	PET/CT	Functional MRI
Available studies	Large prospective independent studies.	Several heterogeneous retrospective studies, in an independent small series of patients, in different disease phases.
Results	Predicts high risk of early progression for patients with residual FLs. Refines the prognosis of patients in conventionally defined CR. Defines the imaging MRD-negative response category. Is complementary to BM cellular or molecular-based techniques. Contributes to defining the sustained MRD-negative response category, associated with the best patient outcomes.	Carries the higher reliability for diffuse BM infiltration. Demonstrated early changes during treatment. Apparently more sensitive than PET with FDG.

Abbreviations: PET/CT, positron emission tomography/computed tomography; MRI, magnetic resonance imaging; CR, complete remission; MRD, minimal residual disease; BM = bone marrow; FLs, focal lesions.

**Table 3:** Validated points and issues to be addressed by on-going trials for imaging-MRD evaluation.

Validated Points
Functional imaging techniques are the suggested tools for the assessment of imaging response. FDG-PET/CT is currently recommended by the IMWG to evaluate MRD after therapy. MRD by NGS or NGF and PET/CT are complementary. Process of the standardization of PET/CT and functional MRI currently ongoing.
Open issues
Optimization of the use of new PET tracers and their correlation with survival outcomes. Correlation of DWIMRI with survival outcomes. Prospective comparison of PET/CT and functional MRI after therapy. Optimal timing of PET/CT and DWIMRI for the evaluation of response and during follow-up. Concordance between imaging and NGS/NGF MRD negativity, at different sensitivity thresholds. Contribution of imaging to the definition and loss of sustained MRD negativity. Impact of MRD assessment on treatment strategies.

Abbreviations: WBLDCT, whole-body low-dose computed tomography; PET/CT, positron emission tomography/computed tomography; MRI, magnetic resonance imaging; SMM, smoldering multiple myeloma; MM, multiple myeloma; SP, solitary plasmocytoma; EMD, extramedullary disease; BM, bone marrow; MRD, minimal residual disease; DWI, diffusion-weighted imaging; NGS, next-generation sequencing; NGF, next-generation flow cytometry; PFS, progression-free survival; OS, overall survival; IMWG, international myeloma working group.
